# Exploring a communication curriculum through a focus on social accountability: A case study at a South African medical school

**DOI:** 10.4102/phcfm.v10i1.1634

**Published:** 2018-05-28

**Authors:** Margaret G. Matthews, Jacqueline M. van Wyk

**Affiliations:** 1 Nelson R Mandela School of Medicine, University of KwaZulu-Natal, South Africa

## Abstract

**Background:**

Good communication is integral to social accountability, and training is included in medical curricula internationally. In KwaZulu-Natal, training is conducted in English, in spite of most public sector patients being mother tongue isiZulu speakers. Communication challenges with patients are common, but good communication and African language teaching are not emphasised in teaching.

**Aim:**

This study explored communication training and how it related to social accountability at a single institution in KwaZulu-Natal.

**Setting:**

This exploratory, qualitative case study design at the medical school explored participants’ perceptions about communication and social accountability and reviewed relevant educational documentation for evidence.

**Methods:**

Purposive sampling was used to select medical students, educators and stakeholders from the educational and service platforms. Focus group discussions and semi-structured interviews were conducted. The data were thematically analysed with reference to Boelen’s social obligation scale for medical schools.

**Results:**

Good communication was valued, but often poorly role-modelled. Participants agreed that communication and isiZulu teaching were insufficiently supported to respond adequately to the needs of local communities. Social accountability was not well understood by students, while medical school educators and other stakeholders indicated that, despite aspirations, this goal had not yet been achieved.

**Conclusions:**

Learning isiZulu language and culture in an integrated manner in both pre-clinical and clinical phases would improve communication with patients, contribute to socially responsive health care, and better address health care needs. Incorporating a social accountability framework in curriculum review would highlight the importance of measuring health outcomes and community impacts, and so enhance the educational mission of the medical school.

## Background

The teaching of clinical communication is an important part of medical training and compulsory in most curricula worldwide.^1,2,3,4^ To be able to communicate effectively, the curriculum needs to expose students to various aspects of communication so that they can understand that effective communication with and beyond the patient (e.g. with family members, carers or other professionals) in health care is vital to good practice.^5^ The integration of communication process skills with clinical content knowledge as part of the clinical method contributes to clinical reasoning in the consultation.^6^ Benefits to good communication include an improved doctor–patient relationship, greater patient and doctor satisfaction, better health outcomes and a reduction in litigation.^7,8,9^

Historically, Africa has adopted medical curricula from the West, but, because of contextual challenges, education on the continent has failed to keep up with international advances, possibly contributing to a perpetuation of health disparities.^10^ Research in African contexts has highlighted noteworthy deficiencies in how doctors are educated with regard to the social contract between medicine and society.^11^ In general and in the South African (SA) context, there is a call to align competencies of medical practitioners with patient, population and health system needs in response to challenges of the 21st century.^12,13,14,15^ South Africa has a diverse multicultural population, with 11 official languages and increasing inequality, including in access to and quality of health care.^16,17^ Recommendations to improve medical education are to reorientate medical curricula to promote transformative learning and for schools to produce graduates who are willing to serve as social change agents with the competencies to address the health needs of the SA populace, including patients from underserved rural areas.^13^

South African health professions (HP) educators have adopted aspects of international guidelines to teach communication in medical curricula.^18,19^ However, clinician educators and students have continued to report communication difficulties between health care workers and non-language concordant patients, and challenges in the teaching and learning (T&L) of communication and language.^19,20,21^ With increasing calls to decolonise HP curricula in SA, some educators and students assert that the forces of imperialism, colonialism and apartheid have shaped HP curricula and resulted in serious inequities that do not serve the interests of the majority black population.^22^ In response, universities are increasing their numbers of staff and students from previously disadvantaged backgrounds, and expanding their offerings of indigenous African language courses.^23^ Nonetheless, a perception lingers that teaching communication and language in health care may be a luxury, especially when faced with the challenges to deliver health care in the over-extended and under-resourced health system. Given the lack of literature describing communication T&L that is socially accountable to communities served by SA medical schools, this study was conducted to explore the topic and inform the discourse on the training of communicators appropriate for local needs.

Following a landmark study, Frenk et al.^14^ recommended adopting a competency-based curriculum to improve educational outcomes, providing a definition of a competency as ‘the habitual and judicious use of communication, knowledge, technical skills, clinical reasoning, emotions, values, and reflection in daily practice for the benefit of the individual and community being served’.^14^ The Undergraduate Education and Training Committee of the Medical and Dental Board of the Health Professions Council of South Africa (HPCSA) similarly recommended the use of a core competencies framework for medical training^24^ based on the CanMEDS model.^25^ This framework, which includes the role of communicator as one of seven critical competencies, was adopted by the College of Health Sciences in 2012.^26^ With reference to the communicator role, the framework resembles Boelen’s concept of the ‘5-star doctor’, which advocates for the conceptualisation and production of doctors with optimal attributes to best respond to the needs of people and societies.^27,28^

Additionally, the HPCSA has recommended the use of a social accountability framework for the accreditation of curricula of medical schools^28^ in an effort to improve social accountability.^11,29,30^ Social accountability refers to the obligation of medical schools to direct education, research and service to address the priority needs of the communities that they serve.^29^ The rationale asserts that medical schools shape the health care system and in response are shaped by it. It further asserts that medical schools, in meriting taxpayer’s support, should fulfil their obligations to society by offering programmes that will positively impact on the health of communities served by them. The use of social accountability frameworks thus holds medical schools accountable to society and increasingly drives the creation of relevant health systems based on people’s needs.^31^

In the domain of education, the concept of socially just pedagogy emphasises the use of curricula that are transformational and that result in transformative education for individual learners.^32,33,34,35^ Indicators for social accountability of medical schools refer specifically to educational programme and outcomes, and also to engagements, governance, faculty and research. Educational outcomes are intended to guide students’ understanding of personal and population health with additional emphasis on the development of professionalism and competence in communication.^36^ Boelen et al. describe the steps towards achieving social accountability using a social obligation scale (refer Table 1) to consider how medical schools meet their obligation as espoused by their educational missions.^36^ Recommendations for a socially accountable programme include the use of teaching methods such as active learning through exposure to the social determinants of health in a longitudinal learning experience. Students should be able to ‘seek information, formulate hypotheses, suggest courses of actions, share views with others, obtain feedback from tutors and take enlightened decisions’.^36^ These abilities are intrinsic in the communicator role, in which students are required to gather, process and share relevant contextual information in a manner that integrates process successfully with content.^5,6,37,38,39^

**TABLE 1:** Levels of a social obligation scale within the educational mission of a medical school.

Level	Criteria
Social responsibility	Committed to what faculty considers being the welfare of society. Educational programmes are community-orientated with institutional objectives defined by faculty. Aims to produce good practitioners based on an implicit identification of society’s health needs. Process is internally assessed.
Social responsiveness	Responds to society’s welfare by directing education, research and service activities towards explicit priority needs. Educational programmes are community-based and institutional objectives based on data. Aims to produce graduates with specific professional meta-competencies to address society’s needs. Outcomes are externally assessed.
Social accountability	As for the socially responsiveness, but also identifies social needs anticipatively, works collaboratively with governments, health systems and the community to positively impact health. Educational programmes are contextualised and institutional objectives defined with society. Aims to produce ‘change agents’ with the capacity to engage with social health determinants and positively impact the health system. Impact is assessed by health partners within the health system.

*Source*: Based on Boelen C, Dharamsi S, Gibbs T. The social accountability of medical schools and its indicators. Educ Health. 2012;25(3):180–194. https://doi.org/10.4103/1357-6283.109785

**FIGURE 1:**
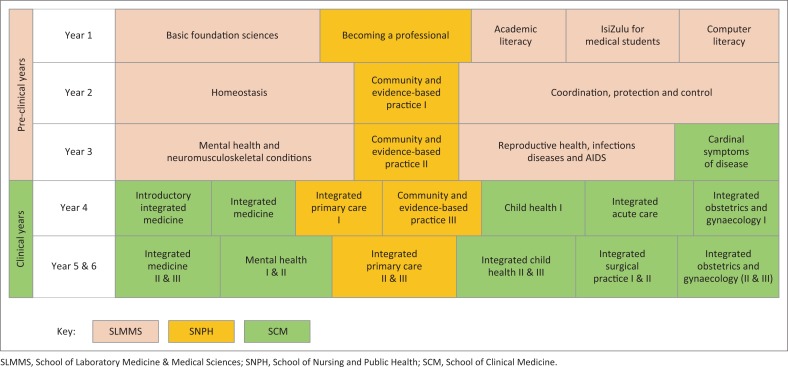
Diagrammatic representation of MBChB programme template years 1–6 (not to scale).

Communication T&L in the MBChB curriculum at the University of KwaZulu-Natal (UKZN) was introduced formally at the undergraduate level in 2010 using a patient-centred model.^39^ Three schools share the responsibility for the delivery of the 6-year curriculum. The School of Laboratory Medicine & Medical Sciences (SLMMS) is responsible for the pre-clinical phase, the School of Clinical Medicine (SCM) for the clinical phase and the School of Nursing & Public Health (SNPH) has responsibilities across both phases (see Figure 1). Communication lectures are covered by behavioural medicine, family medicine and clinical skills. Practical sessions with simulated patients in English are offered within clinical skills training in the first to third years, and communication is also included in the integrated primary care modules in the fourth to sixth years. Small-group teaching and case studies based on social contexts and challenges in the KwaZulu-Natal (KZN) setting inform the teaching strategies that aid students’ understanding of their future roles as HPs. The importance of reflection, both on the patient’s context and students’ own behaviours, is emphasised to assist their understanding and development of the professional meta-competencies. IsiZulu is offered in first year to non-isiZulu medical students who fail a proficiency test. After this module, no formal teaching of isiZulu is offered in the curriculum. In response to students’ reported language challenges and supported by the university language policy, a series of online video scenarios of doctor–patient encounters is offered to facilitate students’ learning of isiZulu communication.^19,40^ These strategies are intended to support students for their clinical encounters with the nearly 80% isiZulu-speaking patient population^41^ with whom they interact in their senior years.

This article describes a study that explored how communication and language skills are taught at an SA medical school (‘the institution’) and how that relates to the social accountability of the school. The specific objectives were to explore stakeholders’ perceptions of the benefits and challenges in communication T&L in the context of the KZN health care environment and the extent to which the criteria for social accountability were expressed in the medical curriculum.

## Method

The method employed in the study is described against the consolidated criteria for reporting qualitative research.^42^

The primary researcher is a clinician who has been involved in teaching communication to medical students in clinical skills, and who has played a role in the development of isiZulu clinical communication. This interest stemmed from past experience as a doctor working in rural practice in an area in which most patients were isiZulu-speaking. The co-author is an educationist and qualitative researcher. Both researchers are women with experience in HP educational research. The participants from the medical school were known to the researchers as colleagues involved in medical education in the same programme. The participants from the rural platform were introduced to the researchers and the study through colleagues involved in the rural training programme. The students had been taught by the primary researcher in their pre-clinical years, but were in final year at the time of the study.

### Study design

The study represents an exploratory, qualitative, educational case study in the interpretivist paradigm that allows for the exploration and understanding of stakeholders’ views. Curriculum, in this paradigm, is viewed as a continuously developing phenomenon that is shaped by the interaction between educators and students and the learning environment, which could be the classroom or broader service platform.^43^ Authentic language use is encouraged and learning is facilitated through reflection and interrogation of local contexts.^22^

### Setting, sampling and participants

Focus group discussions (FGDs) and semi-structured interviews (SSIs) were conducted in 2015–2016 at the medical school, except for one interview that was conducted in the hospital manager’s office for convenience. The study population comprised stakeholders from the medical school and rural teaching platform in KZN Health.

Participants were purposively sampled to each FGD. For the student group, a final year student was asked by the primary researcher to gather a diverse group of his peers. For the educator groups, academic leaders and year or module coordinators from all three schools were invited to participate through an invitation disseminated by the Office of the Dean of T&L, for their knowledge of the content and approach to T&L in the curriculum. The participants from the service platform were purposively sampled from hospitals where students are placed during training.

The final study sample consisted of:

- students in the final year of study (FGD 1, *n* = 5)
- academic staff involved in teaching of undergraduates in three schools in the MBChB programme, viz. SCM (FGD 2, *n* = 9) and SNPH and SLMMS (FGD 3, *n* = 7)
- a rural family medicine practitioner (SSI1), a rural hospital manager (SSI2) and a Department of Health (DoH) representative (SSI3) (*n* = 3).

The participants in the three FGDs were diverse in terms of gender, language, culture and religious beliefs. All groups included men and women, and first languages were English, isiZulu and isiXhosa. The religious beliefs of the sample included Hinduism, Christianity and Islam. All three participants in the SSIs were women, with two being English first language and one Sesotho first language.

The educator group included representatives from all three schools involved in the MBChB programme. The academic positions of the educators included five lecturers, eight senior lecturers, a principal programme officer (SLMMS) and two academic leaders (SCM and SLMMS), one of whom was a senior lecturer and the other an associate professor (*n* = 16).

The participants who represented the service platform included a rural family medicine practitioner and a hospital manager (a clinician in practice), who worked in hospitals where students complete in-service learning, and a DoH representative. The latter participants provided perspectives of the service platform and rural health training component, and could give an external opinion of outcomes of medical students’ training.

### Data collection

Participants received verbal and written information on the nature and purpose of the study. Individual consent was obtained, and permission given for audio recordings of the interviews. Anonymity was assured with data reported under focus group (FG) participant number, SSI number and first language (L1).

The primary researcher facilitated the FGDs, while an assistant served as an observer and recorded field notes. All discussions were conducted in person, except for SSI1, which was conducted telephonically. An interview guide (appended) served to initiate the discussions. Accuracy of content was checked with participants during the interviews and data saturation achieved by offering an opportunity for additional opinions or final comments. The duration of the FGDs was 1–1.5 h, and of the SSIs 35–45 min. The discussions were audio-recorded and professionally transcribed, and checked for accuracy by the researchers against the audio. After the interviews, curriculum documentation was checked to confirm details of T&L methods or activities as well as the use of the HPCSA core competency framework described by the participants. This was to triangulate and verify the data pertaining to curriculum content. This included checking the educational mission statement of the medical school, previous HPCSA reports, programme and module templates and course guidelines.

### Data analysis

Data were analysed using a theoretical thematic analysis as follows:

- Both reviewers initially familiarised themselves with the data.
- The data were then reviewed for content related to the benefits and challenges of communication T&L and exposures, and independently open-coded for subthemes by two coders (the researchers as detailed above).^44,45^
- Themes were reviewed and refined using an iterative process to compare and contrast perceptions of the benefits and challenges in T&L of communication. Perceptions of the other stakeholders were examined for relevance and comparison. A subtheme was included if it was expressed in two or three of the participant groups, namely students, educators and representatives of the service platform. Subthemes were named, and supporting evidence was tabulated under benefits and challenges, for clarity and trustworthiness.

With relevance to the objective that explored the level of social accountability, data from the three participant groups (students, educators and representatives of the rural service platform and DoH) were analysed with reference to the levels in Boelen’s social accountability scale (see Table 1). The data were categorised as either that social accountability was not expressed in the curriculum; that ‘uncertainty’ regarding the concept was expressed; or that an opinion was offered that represented a level of social responsibility, social responsiveness or full accountability. Specific criteria sought were a demonstration of commitment to the welfare of society; whether educational programmes were community-orientated, community-based or defined by society; the type of graduate that the programme was aiming to produce; and whether process, outcomes or impact were internally or externally assessed, or assessed by health partners within the system.

### Ethical considerations

Ethical permission was received from the Humanities and Social Sciences Research Ethics Committee of the University of KwaZulu-Natal and gatekeeper permissions were obtained (HSS 1633/014D).

## Results

### Perceptions of benefits and challenges in communication teaching and learning

In response to the question that explored the benefits of T&L communication, participants in all groups agreed on its value. In particular, student respondents appreciated an understanding of the patient’s perspective, and the content in the pre-clinical phase. They expressed the potential benefit of competent communication as impacting on the doctor–patient relationship and on health outcomes of patients. Educators, especially those in the pre-clinical phase and the SNPH, valued good communication and supported it being taught. The emphasis in the clinical disciplines was, however, on the ‘extraction’ (FGD 2, Participant No 8, L1 English) of disciplinary content rather than on the communication process or the patient perspective. Participants from the service platform confirmed the value of good communication in clinical competence. A disconcerting finding from students’ comments was that competency in communication was neither supported nor role-modelled by many clinical educators. Evidence confirmed that competency in communication was not yet strongly represented by educators in the SCM at tertiary level. Only one student described positive role-modelling by clinical educators at district level.

Clinical educators and the DoH representative believed that good communication between HPs and users of the health system was important in reducing litigation. None of the students mentioned this as a potential benefit.

Language barriers in the clinical environment emerged as a recurrent subtheme across the FGDs and in the interviews. Students, HP educators and participants from the service platform and DoH expressed opinions for isiZulu to be made compulsory, and offered in an integrated vertical manner throughout the curriculum. Even L1 isiZulu students supported this opinion and added that communication T&L had improved their familiarity with terms appropriate to a professional health care context. Cultural barriers were also emphasised by students and educators, while participants from the service platform confirmed the frequency of challenges related to poor understanding of traditional medicine and customary practices.

Participants in both the educator and student groups described how resource challenges had impacted on health care delivery, and cautioned that the resource-constrained environment made ‘good communication’ for HPs a lofty goal. These sentiments were echoed by participants from the service platform, who felt that health care delivery and the burden of disease impacted negatively on patient-centeredness and meaningful communication with patients.

These findings, with the supporting evidence, are tabulated in Table 2 for comparative purposes.

**TABLE 2:** Benefits and challenges in communication teaching in health care.

Themes:	Subthemes	Educational platform		Service platform

		Students (*n* = 5)	Educators (*n* = 16)	Rural family medicine practitioner, hospital manager and DoH (*n* = 3)
Benefits of communication: Teaching and learning/exposure	Important clinical competency	‘Yes, I think it is definitely very important to be taught communication at Medical School.’ (FGD1, Participant No 3, L1 English) Important in ‘making a difference’ and in the doctor–patient relationship (About a patient who had a miscarriage) ‘I was with her at every step of that, like the grieving process and I still…l see her today and I still know her by name. She always greets me when she sees me and I didn’t really think about what an impact I made until it was afterwards because I feel like I… eased the situation for her.’ (FGD1, Participant No 5, L1 isiZulu)	In pre-clinical phase, considered important ‘[*Communication is included*] in the selectives and then in the IPC (Integrated Primary Care) [*modules*] particularly; patient communication has become very central.’ (FGD3, Participant No 6, L1 English) In clinical phase, seen as important as a disciplinary or professional competency related to biomedical content ‘Yes, it is a critical competency but it is an implied competency….being a professional.’ (FGD2, Participant No 5, L1 English) ‘Although we expect students to be able to communicate, I am not sure it is the formal way in which we role play because it is absent in our teaching.’ (FGD2, Participant No 1, L1 English) ‘We are well taught (maybe I come across as cynical) information extraction (not [good] communication).’ (FGD2, Participant No 8, L1 English) ‘It needs to be integrated… otherwise you get the Surgery way of communicating, the Medicine way…’. (FGD3, Participant No 2, L1 English)	Considered important by rural family medicine practitioner, hospital manager and DoH participant: as a competency and in service delivery. Emphasis placed on clinical competencies and in reduction of litigation – see below. ‘Communication is important….even the language, your mannerisms, the tone of your voice.’ (SSI2, Manager, L1 Sesotho) ‘I would want a doctor to communicate well but not necessarily excellently…there is a level of communication you would expect, as a competency…’ (SSI3, DoH, L1 English)
	Benefits to second and first language isiZulu speakers of language and cultural exposures	‘If you had just taken me raw, from the township…. I would have had a lot of difficulties because from some cultures … you say it bluntly as it is. ‘Some words in Zulu, the words I know, I feel like they’re too vulgar to say to a patient.’ (FGD1, Participant No 4, L1 isiXhosa) ‘My home language is Zulu and I say that shamefully because I still have problems communicating in Zulu.’ (FGD1, Participant No 5, L1 isiZulu)	‘There was a Zulu-speaking young woman who went to a rural hospital and said “I have learnt so much about Zulu history and culture that I had no idea about”.’ (FGD3, Participant No 6, L1 English) ‘Specifically from a rural health point of view, [students] write these reflective journals and they come up with really profound things they are experiencing, and things that are so different -“disorientating dilemmas”.’ (FGD3, Participant No 2, L1 English)	‘….learning Zulu is extremely valuable.’ (SSI1, Fam Med, L1 English)
	Reduction in litigation	Not mentioned by students	‘People are learning the value of communication…in the context of malpractice and complaints which is a big thing with the Department of Health.’ (FGD2, Participant No 3, L1 English) ‘… what we are seeing now, from the litigation aspect, we are seeing an enforcement both by the health departments, both in written and verbal communications that we need to improve communication because it does impact in the long term on the profession.’ (FGD2, Participant No 4, L1 English)	‘Litigation against the DoH has increased exponentially over the past few years. I’m not sure what percentage… is due to poor communication on the part of doctors. In my limited experience, it’s often due to factors beyond the doctor’s control… but it’s also due to neglect, not performing clinical functions properly, and….in the form of note-taking (written communication) there is a major deficiency.’ (SSI3, DoH, L1 English)
Challenges in communication	Language and cultural barriers	‘…trying to bridge the language gap….’ (FGD1, Participant No 5, L1 isiZulu) ‘Well, I’m Xhosa … so most of the time I try my best to avoid patients that require me to speak their language.’ (FGD1, Participant No 4, L1 isiXhosa)	‘For instance if a patient is of a different language [*or*] a different economic stature [*or*] a different ethnicity, the levels of communication do differ and you can see that markedly in clinical approaches.’ (FGD2, Participant No 4, L1 English)	‘We have this language barrier…the non-Zulu students – they struggle.’ (SSI2, Manager, L1 Sesotho) ‘For so many years, I’ve been hoping to God that somebody would come…. and help me explain to somebody that they’ve got terminal cancer, but I usually have to do it with sign-language and my form of Zulu. [*Interpreters*] would be lovely.’ (SSI, Fam Med, L1 English) (Regarding customary differences between language groupings) ‘My husband will always say, “How could you say that to a man? In Zulu, we men don’t do that!”.’ (SSI2, Manager, L1 Sesotho) ‘…you know, quite a lot of our problems are traditionally-based…because of traditional meds.’ (SSI1,Fam Med, L1 English)
	Time and resource constraints	(So people become desensitised to [*good communication*], just by the nature of the work?) ‘Yes, because there’s too much… to do and there’s not enough health care staff.’ (FGD1, Participant No 4, L1 isiXhosa)	‘We have done a study where sixty five of our patients have had some elements of domestic abuse, but we never get time to ask that because you have a hundred patients you need to clear in a day…honestly I think theory and practice at the moment are two different things.’ (FGD, Participant No 8, L1 English)	‘If you’re put somewhere ….on a skeleton staff and you’re out of your depth and confused and bewildered, and there are people dying…[*and*] you’ve got resource constraints, I think that does damage to the time and quality of your communication with patients because it’s a matter of survival.’ (SSI1, Fam Med, L1 English)

DoH, Department of Health; SSI, semi-structured interviews; FGD, focus group discussion.

### Social accountability as expressed in the medical curriculum

In analysing the educational mission, the institution expressed its responsibility to society as follows: ‘Our School will be … connected with our stakeholders and community. … We will build credibility and earn global recognition for innovative solutions to health problems’.^46^ Interviews with staff confirmed their awareness of senior management’s engagement with policymakers at national, regional and local levels in attempts to positively impact health.

In contrast to educators, students had a poor understanding of social accountability, but were aware of the social change agent concept and how it related to making a difference within society.^47^ ‘I think you don’t really notice that you’ve made a difference until you’ve made the difference’. (FGD, Participant No 5, L1 isiZulu)

For example, an educator opined that training still concentrated on Western models of disease with little attention to context, stating, ‘We need to understand that … we are providing a largely Western basis [*in HP education*] We need to contextualise it’. (FGD2, Participant No 4, L1 English)

Some educators expressed a commitment to social responsibility, which implies a commitment to what faculty implicitly regards as the welfare of society. This is mainly demonstrated through involvement in community-orientated programmes. Nonetheless, misgivings were expressed about the practicalities of implementing a socially accountable programme.

‘….our registrars and our consultants are doing it but I don’t think we are doing what we are supposed to do and it is not something that can be done overnight. It has to go hand in hand with health care reforms and we are slowly getting there, I mean we are finally trying to change that HIV burden, we are getting ARVs *etc*. We know about accountability and we are trying. I don’t think it is purely because of lack of effort and that we don’t know what we are supposed to be doing. We need to look at everything holistically, even when we are inundated with patients: you can’t do preventive and promotive care that well.’ (FGD2, Participant No 2, L1 English)

Perceptions from other educators indicated that the programme was falling short in supporting the principles underlying social accountability. With reference to students’ role and placement they mentioned:

‘In terms of transformative leaders…. Somehow we are not saying this is the impact that med [*sic*] students can make, that you as a doctor, you went to a rural hospital and you saw you could make a difference. Somehow we are not putting that across, that in fact you can, make a difference.’ (FGD3, Participant No 4, L1 English)

With reference to society:

‘I just want to say that if we are looking at social accountability and access to health care… I think in our curriculum we are shifting to that direction where we are actually going out to provide care to underserved communities. So we are responding to social accountability and access…, but we are only just getting there.’ (FGD2, Participant No 3, L1 English)

When exploring the degree of social accountability as expressed in the educational programme with educators, evidence showed that the institution was delivering a programme that, in principle, supported the production of graduates with specific meta-competencies^26^ to address society’s needs. This is indicative of aspiring to a level of social responsiveness. Some modules included content premised on community-orientated interventions that are defined by faculty. An example was the ‘Making a Difference’ project in the Becoming a Professional module.^48^ In the Selectives modules, students are given research tasks identifying priority community needs. The Integrated Primary Care III module promotes students’ reflections on meta-competencies and social obligations through the use of a portfolio. These processes were, however, internally rather than externally assessed, which is consistent with a level of social responsibility. The demonstration of social responsiveness would require outcomes to be externally assessed.^36^ The analysis revealed no evidence or any attempts to measure the outcomes and impacts of the programme within the health system or on the community. This is a necessary prerequisite for an institution that wants to achieve social responsiveness or true social accountability.^36^

‘It will take a long time to see what impact it has as well.’ (FGD3, Participant No 3, L1 English)

Participants from the rural service platform and DoH expressed a degree of negativity and uncertainty in their responses, and some expressed that social accountability was beyond their level of responsibility. In response to a question asking whether the programme fulfilled the criteria for social accountability, the following responses were given:

‘No, we haven’t come to that yet. We haven’t. We are still trying….We are still training these doctors who are going out there to treat disease. There is nothing wrong with training specialists… but 99% of [*students*] are out to be specialists. They haven’t started thinking I am part of a community. This community needs me.’ (SSI2, L1 Sesotho)

‘Possibly. I don’t know. I don’t know what to measure it against or how one would judge that. …. what does it actually mean?’ (SSI1, L1 English)

‘(Social accountability)…is a complex topic and it’s quite dangerous for the DoH to become too immersed in social accountability, in a sense that we cannot deflect from our primary obligation which is to provide health care.’ (SSI3, L1 English)

## Discussion

The study explored the T&L of communication of medical students in relation to the social accountability framework. It explored participants’ views of the benefits and challenges in communication T&L and the degree to which community needs had been considered in curriculum design.

Communication has been identified as important in promoting health care that is socially accountable. This study highlighted the benefits of good communication, and of communication training in medical curricula. It showed that good communication was valued as a clinical competency by students, educators and stakeholders representing the service platform. Students appreciated the difference that communicating using a patient-centred approach had made in relationships with their patients. Educators acknowledged its importance and felt that a generic approach to good communication should be integrated into disciplinary teaching, an important basic principle in communication teaching.^6^

Other benefits included an appreciation of how language in communication benefitted both second and first language isiZulu speakers, allowing them a better appreciation of cultural influences in health.^49^ Educators and service platform participants linked better communication with reduced litigation.^50^

Regarding the challenges experienced, subthemes related to language and cultural barriers and resource constraints, which result in significant challenges in education and service delivery. While studies show that the provision of health services in the language of patients supports social accountability and service delivery,^51,52,53^ the evidence shows that many graduates of this medical school continue to experience language and cultural barriers. To compound this problem in most cases, there are no qualified interpreters available, and interactions are often mediated by other members of staff,^54^ thus impacting on their primary responsibilities. This potentially contributes to poor service delivery and possibly to increased litigation in the public health care sector in KZN.

For graduates to be able to communicate in isiZulu, the commonest language in KZN, vertical integration of isiZulu language and cultural learning through the programme would best be combined with explicit horizontal integration within the clinical disciplines, with demonstration of competency in communication in both English and isiZulu deemed essential for professionalism and clinical competence of graduates. We recommend that a patient-centred approach that considers the needs of each patient and respects language and cultural differences should be taught by educators and strongly supported and role-modelled by clinical service staff. Students should be made aware of litigation and its causes, including the role of poor communication.^50^ Ideally, there is a need for an institutional culture that promotes good values, excellence in communication and social accountability, integrated in clinical teaching,^27^ thus exposing students to a coherent model throughout their training.

Social accountability principles are regarded as important by the World Health Organization in the accreditation of medical schools. The concept requires that the interactions of the medical school with its community be grounded in societal needs.^28^ It also presumes that graduates from such programmes will have achieved the necessary competencies to be utilisable immediately in the health system. Despite initiatives to improve the social accountability of the programme by directing education, research and service activities towards the needs of society,^36^ evidence from this study has shown that these principles were not coherently expressed throughout the programme. The institution’s aspirations to be socially accountable and to produce graduates with the requisite competencies were also not affirmed. The concepts were not emphasised consistently, nor had the requisite competencies to make graduates fit for purpose been adequately assessed. There was no evidence that a formal framework for social accountability had been adopted or that outcomes and impacts relating to health were being considered or measured. Common shared values on social accountability between the role players were not yet evident. Role players appeared to conceptualise their roles differently, with an emphasis placed on delivering mainly curative services in a challenging and resource-constrained environment.

**FIGURE 2:**
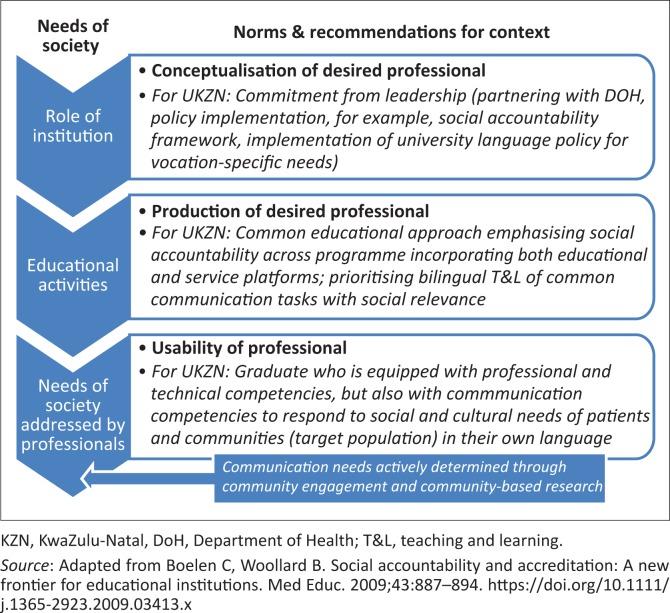
The conceptualisation–production–usability model: Recommendations for University of KwaZulu-Natal context.

The conceptualisation–production–usability (CPU) model in social accountability refers to the development of a partnership between the educational institution and the health system that could be achieved through establishing a shared and common set of values between the stakeholders.^28^ The role of the institution lies in conceptualising the norms for the desired professional and in directing educational activities towards the production of such a professional. The necessary qualities of such a professional have been described in various models^24,25,27^ that emphasise the moral and social obligations of doctors to society. The competency-based approach asserts that the competencies acquired during training should be appropriate for the future working environment.^14^ With the role of communicator considered an essential competency, it is evident that the language requirement, an important priority for graduates, remains unresolved. To be a competent communicator and to improve doctors’ usability in the health system, such a doctor should be sufficiently skilled to provide clinical care especially at primary level to isiZulu-speaking patients (Figure 2).

Context-facilitated language learning with an increased emphasis on primary health care and longitudinal exposures to communities in KZN would be aligned with Boelen’s social accountability framework^36^ and its ultimate aim to improve service delivery.^36^ Placement in the rural context, where exposures to isiZulu-speaking patients, traditional medicine and cultural practices are common, would build on the isiZulu communication skills covered in the pre-clinical phase. Immersion in the community^55^ would also improve students’ understanding of how patients access health care in KZN, moving between traditional and Western models. It would provide students with the opportunity to experience and critically reflect on patients’ needs to access health care in a language that can convey the necessary cultural nuances.^49,56^ Providing feedback would be important to develop self-awareness, desirable behaviours and attributes such as empathy and respect for others,^5^ and to promote transformative learning, which educators linked with these experiences (see Table 2).^34^ Such exposures would facilitate students’ understanding of the impact of major social determinants, such as poverty and inequality, and sensitise them to their social responsibilities as HPs. The incorporation of both humanistic and systemic principles includes consideration of the needs of the most vulnerable in society, and demonstrates the need for stakeholders to partner in this endeavour for maximum impact.

Operationalising the use of the social accountability framework would require commitment from leadership to implement policy, and a common approach across the MBChB programme. Implementation with a high level of input from medical students and community stakeholders would promote inclusivity, deepen students’ understanding of their roles and determine priorities for improving communication with patients and communities, simultaneously responding to calls to decolonise and transform the curriculum. Research is important to assess process, outcomes and particularly the impact on the public good.

### Limitations

As an exploratory case study in one medical school, the findings are not generalisable to other medical schools. The findings report participants’ perceptions of the degree of social accountability expressed in the MBChB programme and the communication challenges related to indigenous language use and other factors in SA health care. The study, however, provided rich data in the local context that can generate discussion and contribute to the body of knowledge on T&L communication and social accountability of SA medical schools.

## Conclusion

This study has highlighted the perceived benefits of good communication and challenges posed by communication barriers in a medical programme. Recommendations are made to incorporate a social accountability framework with an emphasis on isiZulu language and cultural learning for the communicator role in a clinical context. This would contribute to the social accountability of the institution, the production of graduates with better usability in the health system, and guide the development of a socially accountable and relevant communication curriculum based on specific local needs.
